# Abdominal Obesity and Brain Atrophy in Type 2 Diabetes Mellitus

**DOI:** 10.1371/journal.pone.0142589

**Published:** 2015-11-11

**Authors:** Rachel E. D. Climie, Chris Moran, Michele Callisaya, Leigh Blizzard, James E. Sharman, Alison Venn, Thanh G. Phan, Richard Beare, Josephine Forbes, Nicholas B. Blackburn, Velandai Srikanth

**Affiliations:** 1 Menzies Institute for Medical Research, University of Tasmania, Hobart, Tasmania, Australia; 2 Stroke and Ageing Research Group, Monash Medical Centre, Monash University, Clayton, Victoria, Australia; 3 Aged Care, Caulfield General Medical Centre, Alfred Health, Melbourne, Victoria, Australia; 4 Developmental Imaging, Murdoch Children’s Research Institute, Melbourne, Victoria, Australia; 5 Mater Research Institute, University of Queensland, TRI, Brisbane, Queensland, Australia; Harbin Medical University, CHINA

## Abstract

**Aim:**

Type 2 diabetes mellitus (T2D) is associated with gray matter atrophy. Adiposity and physical inactivity are risk factors for T2D and brain atrophy. We studied whether the associations of T2D with total gray matter volume (GMV) and hippocampal volume (HV) are dependent on obesity and physical activity.

**Materials and Methods:**

In this cross-sectional study, we measured waist-hip ratio (WHR), body mass index (BMI), mean steps/day and brain volumes in a community dwelling cohort of people with and without T2D. Using multivariable linear regression, we examined whether WHR, BMI and physical activity mediated or modified the association between T2D, GMV and HV.

**Results:**

There were 258 participants with (mean age 67±7 years) and 302 without (mean age 72±7 years) T2D. Adjusting for age, sex and intracranial volume, T2D was independently associated with lower total GMV (p = 0.001) and HV (p<0.001), greater WHR (p<0.001) and BMI (p<0.001), and lower mean steps/day (p = 0.002). After adjusting for covariates, the inclusion of BMI and mean steps/day did not significantly affect the T2D-GMV association, but WHR attenuated it by 32% while remaining independently associated with lower GMV (p<0.01). The T2D-HV association was minimally changed by the addition of BMI, steps/day or WHR in the model. No statistical interactions were observed between T2D and measures of obesity and physical activity in explaining brain volumes.

**Conclusions:**

Abdominal obesity or its downstream effects may partially mediate the adverse effect of T2D on brain atrophy. This requires confirmation in longitudinal studies.

## Introduction

People with type 2 Diabetes Mellitus (T2D) are at high risk of developing cognitive impairment [1] and dementia [2]. We have recently shown that T2D is associated with lower total gray matter volume (GMV) and that GMV loss may explain the association between T2D and cognitive dysfunction [3]. However, the pathways leading to loss of GMV in T2D are unknown.

Obesity and physical inactivity are commonly seen in people with T2D, and have also been associated with brain atrophy [4–8] and dementia [9, 10]. The distribution of body fat may also play a role in explaining these associations. In particular, abdominal adiposity is linked to chronic inflammation and reduced insulin sensitivity [11], both potentially important factors in determining neuronal health [9, 10]. In support of this concept, a recent imaging study demonstrated that visceral fat accumulation was associated with reduced cortical thickness independent of BMI [12]. Low levels of physical activity [8] or cardiovascular fitness [13], which are determinants of low grade inflammation, vascular health and metabolic health [14] have also been associated with lower GMV.

The roles of obesity and physical activity in determining gray matter loss in people with T2D have not been studied. Since these are modifiable risk factors, a better understanding of their relative contributions to brain health in T2D will help guide interventions aimed at preserving cognition in people with T2D who represent a high-risk group for developing dementia. We hypothesised that the association between T2D and GMV will either be modified or mediated by measures of obesity or physical inactivity.

## Materials and Methods

### Study sample

The sample consisted of participants recruited into the Cognition and Diabetes in Older Tasmanians study, the recruitment details of which have previously been described [3]. Those with T2D were selected from the National Diabetes Service Scheme (NDSS) register if aged >55 years and living in the Southern Tasmanian postcodes 7000–7199. The NDSS is managed by Diabetes Australia and provides information and support for individuals with diabetes who enroll voluntarily. The diagnosis of T2D within NDSS is based on physician assessment using standard criteria including; fasting plasma glucose ≥7. 0 mmol/L, random plasma glucose ≥11.1 mmol/L, or 2 hour glucose ≥11.1mmol/L post oral glucose tolerance test. The population-based comparison group consisted of individuals who were aged ≥60 years without T2D randomly selected from the same Southern Tasmanian postcodes (7000–7199) into the Tasmanian Study of Cognition and Gait [3]. The absence of T2D in the comparison group was determined by the following; fasting plasma glucose <7.0mmol/L, random plasma glucose <11.1mmol/L and HbA_1c_ ≤6.5% (48mmol/mol) in those individuals without a history of T2D. All potential participants received invitation letters followed by telephone contact for enrolment into the study. Excluded were people living in a nursing home and those with any contraindication to magnetic resonance imaging (MRI). The Southern Tasmanian Health and Medical Human Research Ethics Committee and the Monash University Human Research Ethics Committee approved the study and all participants signed informed consent.

### Measurements

Standardised questionnaires were administered to obtain demographic data, clinical information about the duration of T2D, years of formal education, health and medical history including that of cardiovascular disease and risk factors, and medication use. The 15-item Geriatric Depression Scale (GDS) [15] was used to determine mood.

#### Obesity

Waist and hip circumference were measured in duplicate unless there was a difference of more than two centimeters between the first and second measurement, in which case a third measurement was taken and the average of all three measures was used in the analysis. Waist-hip ratio (WHR) was calculated as a measure of abdominal obesity dividing waist circumference (cm) by hip circumference (cm). Height (m) and weight (kg) were measured and body mass index (BMI) was calculated as weight divided by height squared.

#### Physical activity

Daily physical activity was measured using a Yamax pedometer. Participants were instructed to attach the pedometer to the waistband of trousers/skirt above their dominant leg and to wear the pedometer for 7 consecutive days, whilst going about normal daily activity. They were instructed to reset the pedometer at the start of every day and to record the number of steps displayed on the monitor in a pedometer diary at the end of each day. Mean steps/day were calculated by dividing the total number of steps on days where the participant wore the pedometer for ≥eight hours a day, by the number of days that the pedometer was worn. In a sub analysis (n = 115) we determined that a cut off value for wear time of ≥eight hours a day would result in 95% of mean steps/day being captured.

#### Brain MRI

MRI brain scans were performed using a 1.5T General Electric Signa Excite T scanner with sequences as follows: High-resolution T1 weighted spoiled gradient echo (TR 35ms, TE 7ms, flip angle 35°, field of view 24 cm, voxel size 1 mm^3^) comprising 120 contiguous slices; T2 weighted fast spin echo (TR 4300 ms; TE 120 ms; NEX 1; turbo factor 48; voxel size 0.90 x 0.90 x 3 mm); FLAIR (fluid attenuated inversion recovery) (TR = 8802 ms, TE = 130 ms, TI = 2200ms, voxel size 0.50 x 0.50 x 3 mm); GRE (TR0.8ms, TE 0.015, flip angle 30°, voxel size 0.9 x 0.9 x 7 mm). All processing and segmentation steps were performed by investigators blinded to T2D status. The scans were registered to a standard 152 brain Montreal Neurological Institute template in stereotaxic coordinate space. Gray and white matter were automatically segmented using methods in statistical parametric mapping software SPM5 [16]. Hippocampi were manually segmented using standard methodology and landmarks with high test-retest reliability [17]. Total GMV and hippocampal volume (left, right and total HV) were calculated using standard in-house voxel counting algorithms.

#### Blood biochemistry and genotyping

Following an overnight fast, venous blood samples were taken from the antecubital fossa. Analytical biochemistry of fasting plasma glucose, glycated haemoglobin (HbA_1c_), insulin, lipid profile and C-reactive protein (CRP) were performed at the Royal Hobart Hospital, Tasmania, Australia using accredited laboratory techniques. We also measured serum levels of tumor necrosis factor alpha (TNFα) and interleukin 6 (IL6) using Multiplex Bead Arrays (Lincoplex, Linco Research Inc. Missouri, USA). Whole blood DNA extraction and apolipoprotein ε4 allele (*APOE*-ε4) SNP genotyping (rs429358 and rs7412) using Sequenom MassArray iPLEX technology.

#### Other clinical measures

Mean systolic blood pressure was taken from three consecutive seated brachial blood pressure measurements from the right arm of each participant using an Omron M4 sphygmomanometer. Hypertension was defined as systolic blood pressure>140mmHg and/or diastolic blood pressure >90 mmHg and/or current use of anti-hypertension medication. Homeostatic model assessment of insulin resistance (HOMA-IR) was calculated from fasting plasma glucose and insulin levels using the formula (Insulin x Glucose)/22.5 [18]. Hyperlipidaemia was defined as total cholesterol ≥6 mmol/L and/or current use of statin. We also had measures of tissue advanced glycation endproduct (AGE) accumulation available in most participants using the skin autofluorescence technique [19].

### Statistical methods

Independent t tests were performed for continuous variables with normal distributions, Wilcoxon rank sum test for continuous measures with non-normal distributions, and Chi square tests for dichotomous variables while comparing characteristics between patients with and without T2D. Firstly exploratory unadjusted correlations and regressions were conducted adjusting for age, sex and total intracranial volume to examine the association between T2D and cortical volumes (GMV, HV), and associations of obesity and habitual physical activity (WHR, BMI, mean steps/day) with cortical volumes. Multivariable regression models were then used to examine whether the T2D-brain volume relationships were confounded, modified or mediated by measures of obesity and physical activity. To study effect modification, we assessed for an interaction between T2D and measures of obesity and physical activity in explaining brain volumes using a test of significance of the respective product terms (T2D × WHR; T2D × BMI, T2D × mean steps/day), adjusting for age, sex, total intracranial volume, education, *APOE*-ε4 status (grouped as ε4 allele carriers or non-carriers), vascular risk factors (a summary variable coded for the presence of hypertension, and/or hyperlipidemia, and/or smoking, and/or history of stroke, and/or history of ischemic heart disease), years of formal education and GDS score. SAF was used as an additional covariate among participants in whom it was available. To examine potential mediation of the association between T2D and brain volumes, we successively entered mean steps/day, BMI and WHR into multivariable regression models relating T2D to the respective brain volume measure. Mediation was judged to be present, if the addition of the potential mediator (mean steps/day, BMI or WHR) attenuated the β coefficient for the association between T2D and the brain volume measure by >30%, and the β coefficient and standard errors for the mediator remained relatively unchanged from its value without T2D in the model. Finally, we explored the effects of potential mechanistic variables (HOMA-IR, HbA_1c_, and inflammatory cytokines including CRP, TNFα and IL6) by adjusting for them in the final models. All statistical analyses were performed using STATA version 12 (StatCorp.College Station Tx.) and p<0.05 was considered statistically significant.

## Results

The participant characteristics are summarised in **Table 1**. Among a total of 560 participants, There were 258 with T2D (mean age 67 ± 7 years) and 302 without T2D (mean age 72 ± 7 years) with complete data on the primary exposure (obesity measures and mean steps/day) and outcome (brain MRI measures) variables. The median duration of T2D was 6 years (interquartile range 3–11 years). In univariable comparisons against those without T2D, people with T2D had significantly greater BMI, WHR, fasting blood glucose, HbA_1c_, and triglyceride levels, were more likely to report a history of ischemic heart disease, stroke, hypertension, hyperlipidemia, and be on treatment for both (all p<0.05), but had similar mean steps/day.

**Table 1:** Participant characteristics. T2D –type 2 diabetes mellitus, SD–standard deviation, IQR–interquartile range, HOMA-IR–homeostatic model assessment of insulin resistance, MRI–magnetic resonance imaging. ^Hypertension—self-reported history of hypertension or mean systolic blood pressure >140 or mean diastolic blood pressure >90 mmHg. p value is for unadjusted comparisons. Wilcoxon rank sum tests for fasting glucose, HbA_1c,_ triglycerides, HOMA-IR, C-Reactive Protein, GDS score. Independent t-tests or chi-square tests for all other variables.

Variables	T2D (n = 258) Mean (SD) or n (%)	Non T2D (n = 302) Mean (SD) or n (%)	P value
Male sex	159 (62)	161 (53)	0.061
Age (years)	67 (7)	72 (7)	<0.001
Median duration of T2D (years; IQR)	6 (3–11)	-	-
Ischemic heart disease	51 (20)	52 (17)	0.46
History of stroke	22 (8)	20 (6)	0.42
Smoked	140 (54)	155 (51)	0.49
^Hypertension	215 (83)	216 (72)	0.001
Systolic blood pressure (mmHg)	137 (19)	142 (22)	0.005
Diastolic blood pressure (mmHg)	77 (10)	80 (12)	<0.001
Hyperlipidaemia	173 (67)	143 (47)	<0.001
Blood pressure lowering medication	182 (70)	144 (48)	<0.001
Statin use	161 (62)	74 (25)	<0.001
Body mass index (kg/m^2^)	30.0 (4.6)	27.2 (4.0)	<0.001
Overweight (BMI 25–30)	108 (42)	148 (49)	0.076
Obese (BMI>30)	115 (45)	59 (20)	<0.001
Waist-hip ratio	0.96 (0.08)	0.90 (0.08)	<0.001
Fasting blood glucose (mmol/l)	7.7 (2.2)	5.3 (0.55)	<0.001
HbA_1c_ (%)/ (mmol/mol)	7.1 (1.2)/ 54.1	5.6 (0.3)/ 37.7	<0.001
Total cholesterol (mmol/L)	4.4 (1.0)	5.3 (1.2)	<0.001
Triglycerides (mmol/L)	1.7 (0.8)	1.3 (0.6)	<0.001
HOMA-IR (IU)	2.18 (13.30)	5.93 (1.56)	<0.001
C-reactive protein (mg/dL)	3.31(7.44)	3.70 (7.09)	0.53
*APOE*-ε4 allele	70 (27)	72 (24)	0.53
Geriatric Depression Scale (GDS) score	2.2 (2.4)	1.7 (2.0)	0.02
Formal education (years)	12 (4)	11 (4)	0.051
Mean steps per day	6088 (3481)	6201 (3216)	0.67
*MRI Cortical volumes*			
Total gray matter volume (ml)	586.9 (60.2)	582.6 (61.1)	0.40
White matter volume (ml)	457.8 (58.6)	455.15 (55.5)	0.58
Total hippocampal volume (ml)	4.6 (0.8)	5.4 (0.9)	<0.001
Left hippocampal volume (ml)	2.2 (0.4)	2.6 (0.5)	<0.001
Right hippocampal volume (ml)	2.3 (0.4)	2.8 (0.5)	<0.001

### T2D, obesity and habitual physical activity

Unadjusted simple correlation coefficients for T2D with obesity and physical activity measures were as follows: BMI (0.30, p<0.001), WHR (0.33, p<0.001), mean steps/day (-0.01, p = 0.79). After adjusting for age and sex, compared with those without T2D, patients with T2D had significantly greater WHR (β = 0.046, 95% CI 0.03 to 0.06, p<0.001) and BMI (β = 2.30, 95% CI 1.57 to 3.03, p<0.001), and walked fewer mean steps/day (β = –854, 95% CI –1403 to –305, p = 0.002). In the whole sample, greater WHR and BMI were significantly and inversely related to mean steps/day (both p<0.001).

### T2D, obesity, habitual physical activity and brain volumes

Associations of these variables with total GMV and HV are presented in **Table 2**, adjusted for age, sex and total intracranial volume. T2D was significantly associated with lower total GMV (β = –10.04, 95% CI –15.89 to –4.19, p = 0.001), left HV (β = –0.39, 95% CI –0.47 to –0.32, p<0.001), right HV (β = –0.45, 95% CI –0.53 to –0.37, p<0.001) and total HV (β = –0.85, 95% CI –0.99 to –0.70, p<0.001). Greater WHR (p<0.001) and BMI (p = 0.01), and fewer mean steps/day (p = 0.02) were independently associated with lower total GMV. Greater WHR, greater BMI, and fewer mean steps/day were associated with lower left, right HV and total HV (all p<0.05).

**Table 2:** Associations of type 2 diabetes mellitus (T2D), waist-hip ratio (WHR), body mass index (BMI), mean steps/day and cortical volumes (n = 560). β is unstandardized coefficient. CI–confidence interval. All regressions adjusted for age, sex and total intracranial volume.

	T2D β (95% CI)	P value	WHR β (95% CI)	P value	BMI β (95% CI)	P value	Mean steps/day β (95% CI)	P value
Total gray matter volume (ml)	-10.04	0.001	-107.77	<0.001	-0.82	0.01	0.001	0.02
	(-15.89, -4.19)		(-146.81, -68.73)		(-1.47, 0.18)		(0.0001, 0.002)	
Left hippocampal volume (ml)	-0.39	<0.001	-1.48	<0.001	-0.01	0.03	0.00002	0.005
	(-0.47, -0.32)		(-2.03, -0.93)		(-0.01, -0.001)		(5.39^−6^, 0.00003)	
Right hippocampal volume (ml)	-0.45	<0.001	-1.18	<0.001	-0.009	0.05	0.00002	0.002
	(-0.53, -0.37)		(-1.77, -0.59)		(-0.02, -0.00002)		(7.93^−6^, 00003)	
Total hippocampal volume (ml)	-0.85	<0.001	-2.70	<0.001	-0.02	0.02	0.00004	0.001
	(-0.99, -0.70)		(-3.76, -1.64)		(-0.04, -0.003)		(0.00002, 0.00007)	

### Analysis of effect modification and mediation

There were 532 participants with complete data available for multivariable analysis excluding the variable SAF. **Table 3** shows the change in the association between T2D and total GMV (adjusted for age, sex, vascular risk, education, *APOE*-ε4 and GDS score) when each additional factor of interest (i.e. mean steps/day, BMI, WHR) is entered into the models. The addition of mean steps/day (Model 2) and BMI (Model 3) did not appreciably alter the association between T2D and total GMV. The addition of WHR (Model 4) attenuated the association between T2D and total GMV by 32% (compared with Model 3) rendering the T2D-GMV relationship statistically non-significant, while WHR remained independently associated with total GMV (p<0.001), and the standard errors for T2D and WHR remained unchanged. The association between T2D and total HV **(Table 4)** was unchanged by the addition of mean steps/day, BMI and WHR. Greater mean steps/day, but not BMI or WHR, was independently associated with greater total HV (p<0.05). The addition of HOMA-IR, HbA_1c_, CRP, TNFα and IL6, and SAF (available only in 486 participants, data not shown) to the final models (Models 4) for both total GMV and HV did not change the observed associations. There were no significant interactions between T2D and measures of obesity or physical activity in explaining cortical volumes (p>0.05 for all product terms).

**Table 3:** Effects of mean steps/day, body mass index (BMI) and waist-hip ratio (WHR) on the association between type 2 diabetes mellitus (T2D) and total gray matter volume (n = 532). β–beta coefficient, CI–confidence interval, T2D –type 2 diabetes mellitus, BMI–body mass index, WHR–waist-hip ratio. All models adjusted for age, sex, years of education, total intracranial volume, vascular risk (hypertension and/or hyperlipidemia and/or smoking and/or history of stroke and/or history of ischemic heart disease), apolipoprotein ε4 allele and Geriatric Depression Scale score.

Variables	Model 1	Model 2	Model 3	Model 4
	β (95%CI)	β (95%CI)	β (95%CI)	β (95%CI)
T2D	-7.98 (-13.96, -2.01)*	-7.48 (-13.48, -1.45)*	-7.40 (-13.64, -1.16)*	-5.05 (-11.32, 1.22)
Mean steps per day		0.001 (-0.0002, 0.002)	0.001 (-0.0002, 0.002)	0.001 (-0.001, 0.02)
Body mass index			-0.14 (-0.85, 0.57)	0.24 (-0.50, 0.97)
Waist hip ratio				-72.26 (-117.55, -26.97)^

*p<0.05

^p<0.01. Model 1 –association between T2D and total gray matter volume. Model 2 –model 1 adjusted additionally for mean steps/day. Model 3 –model 2 adjusted additionally for BMI. Model 4 –model 3 adjusted additionally for WHR.

**Table 4:** Effects of mean steps/day, body mass index (BMI) and waist-hip ratio (WHR) on the association between type 2 diabetes mellitus (T2D) and total hippocampal volume (n = 532). β–beta coefficient, CI–confidence interval, T2D –type 2 diabetes mellitus, BMI–body mass index, WHR–waist-hip ratio. All models adjusted for age, sex, years of education, total intracranial volume, vascular risk (hypertension and/or hyperlipidemia and/or smoking and/or history of stroke and/or history of ischemic heart disease), apolipoprotein ε4 allele and Geriatric Depression Scale score.

Variables	Model 1	Model 2	Model 3	Model 4
	β (95%CI)	β (95%CI)	β (95%CI)	β (95%CI)
T2D	-0.94 (-1.08, -0.79)^	-0.92 (-1.06, -0.77)^	-0.94 (-1.09, -0.78)^	-0.91 (-1.06, -0.75)^
Mean steps per day		0.00003 (8.67^06^, 0.0001)*	0.00003 (0.00001, 0.0001)*	0.00003 (9.88^06^, 0.0001)*
Body mass index			0.01 (-0.01, 0.03)	0.02 (-0.001, 0.04)
Waist hip ratio				-1.09 (-2.21, 0.03)

*p<0.05

^p<0.01. Model 1 –association between T2D and total hippocampal volume. Model 2 –model 1 adjusted additionally for mean steps/day. Model 3 –model 2 adjusted additionally for BMI. Model 4 –model 3 adjusted additionally for WHR.

## Discussion

We found that the adverse association between T2D and total GMV may be partially mediated by abdominal obesity. Moreover, WHR, but not BMI or mean steps/day, remained independently associated with total GMV. Mean steps/day did not affect the relationship between T2D and total GMV. By contrast, neither WHR, BMI or mean steps/day appeared to affect the association between T2D and total HV. However, across all individuals mean steps/day, but not WHR or BMI, remained independently associated with total HV.

Although previous studies have reported that obesity is associated with lower total brain or regional volumes in the general population [4, 5, 12, 20, 21], none, to our knowledge, have examined these relationships in people with T2D. In our previous study [3], we were unable to demonstrate an independent association of T2D with white matter volume. Therefore we did not explore white matter volume as an outcome, although others have demonstrated that obesity (overall and abdominal) is related to lower white matter volume in morbidly obese people [21]. We found that WHR, but not BMI, explains a large portion of the T2D-GMV association suggesting that abdominal obesity and its related mechanistic factors may be important drivers of gray matter atrophy in T2D. WHR was also independently associated with total GMV. An interpretation of this finding is that T2D confounds the relationship between WHR and GMV, but the stability of standard errors in the models suggests this is less likely. T2D is likely to represent a clinical state further downstream of abdominal obesity in the causal pathway to cortical atrophy. In support of this concept, abdominal adiposity often precedes the development of insulin resistance and T2D [11]. The direction of causality between WHR and total GMV cannot be confirmed based on these cross-sectional analyses alone, because atrophy of brain regions that regulate dietary habits may theoretically explain the observed relationships [22]. However, a Mendelian randomisation analysis in the 3C-Dijon Study demonstrated that the association between WHR and lower total GMV in the general population [4] was likely to be causal. Our results are consistent with cross-sectional and longitudinal data from the general population showing that the associations between obesity and brain volumes are more pronounced for abdominal obesity rather than measures of global body mass such as BMI [4, 5, 23]. Abdominal fat differs in its metabolic activity compared with peripheral fat, is strongly linked to the production of pro-inflammatory cytokines and the generation of insulin resistance [11], and is more strongly predictive of cardiovascular disease than measures of global obesity (e.g. BMI) [24]. Although fewer mean steps/day were associated with T2D as well as total gray matter and hippocampal atrophy, mean steps/day did not explain the T2D-GMV or T2D-HV associations. Interventions involving moderate and vigorous aerobic [25, 26] or resistance training [27] interventions are known to preserve brain structure and function as well as improve glycemic control in older individuals [28]. It is possible that our measure of physical activity was not sufficiently sensitive to capture the exercise intensity and type necessary to influence T2D related brain atrophy. However, similar to recent work [29], we showed that those individuals who engaged in more physical activity had lower WHR. Mean steps/day did remain independently related to total HV, in line with previous studies [30, 31], suggesting physical activity is important for maintaining total HV irrespective of diabetes status.

Chronic low grade inflammation, insulin resistance, advanced glycation endproducts (AGEs), hormonal effects and vascular disease may all be mechanisms that could explain the associations between T2D, abdominal obesity and brain atrophy. The association of T2D and WHR with GMV was independent of inflammatory cytokines in our study, however, peripheral inflammatory cytokine levels are poor measures of neuroinflammation which requires estimation with specialised neuroimaging [32]. Neuronal insulin resistance is associated with impaired amyloid clearance [33] and increased tau phosphorylation in the human brain [34] and in mouse models of T2D [35]. However, adjustment in our final models (Model 4) for HOMA-IR did not alter the T2D-GMV and T2D-HV associations. Finally, the associations of T2D, obesity measures and mean steps per day with GMV or hippocampal volume were independent of SAF, a measure of long-term tissue advanced glycation, although we were unable to adjust for measures of circulating AGEs. Abdominal obesity is also strongly associated with vascular mechanisms that may explain brain atrophy such as arterial stiffness [36, 37]. It is tempting to consider whether interventions targeting abdominal obesity or related factors may protect against brain atrophy in T2D. Lifestyle interventions (such as increased physical activity and decreasing caloric intake) seem a reasonable option although they do not necessarily preferentially target abdominal adiposity [38] and may be difficult to maintain. Bariatric surgery in highly selected morbidly obese middle-aged individuals was shown to be associated with improved cognition in a small study (n = 21), but the contribution of weight loss to this improvement was not explored in relation to other mechanistic variables [39]. There is renewed use of antidiabetic agents such as thiazolidinediones [40] and metformin [41] that have modest effects on abdominal obesity, as well as leptin analogs to determine whether use of these agents may ameliorate cognitive decline in individuals with T2D [42]. Additionally, interventions that target adiposity-related mechanisms such as insulin signaling (e.g. analogs of glucagon-like peptide) deserve further study for preserving brain health in T2D.

Strengths of this study include a large sample size, a robust definition of T2D, quantitative measures of exposures (physical activity, BMI and WHR) and outcome (brain volumes) using validated and standardised techniques, adjustment for several potential confounders, and careful analysis for effect modification and mediation. The following are limitations of our study. Due to the cross sectional design, this study does not permit us to draw conclusions about causality. On the other hand, our findings are consistent with evidence linking abdominal obesity to cognitive decline [43, 44] and brain atrophy in non-diabetic populations [4, 5, 12, 20], and provide a good basis for the longitudinal study of abdominal obesity on brain atrophy in patients with T2D. Secondly, as patients with T2D were recruited based on their willingness to participate in research indicated on their NDSS membership, our sample might be over-represented by healthier individuals with T2D. Nonetheless, we showed consistent and expected differences in anthropometric and biochemical measures between those with T2D and the comparison group, (i.e. patients with T2D had higher WHR, BMI, fasting blood glucose and HbA_1c_). Although pedometers provide an objective measure of habitual physical activity and are simple and inexpensive [45], they do not provide information on sedentary behavior, non-ambulatory physical activity (i.e. swimming or resistance training), intensity or type of physical activity [46]. Finally, the pedometers were only worn for 7 days and, therefore, may not provide a good representation of long-term physical activity.

In summary, abdominal obesity appears to be an important factor in explaining the adverse impact of T2D on total GMV and these results require confirmation in longitudinal studies. In people with T2D, who represent a high-risk group for developing dementia and cognitive dysfunction, interventions targeting abdominal obesity or its related downstream factors may present promising avenues for reducing the risk of T2D related total GMV atrophy.

## Supporting Information

S1 Dataset Minimum dataset. (CSV)
